# A multi‐omic study reveals *BTG2* as a reliable prognostic marker for early‐stage non‐small cell lung cancer

**DOI:** 10.1002/1878-0261.12204

**Published:** 2018-05-04

**Authors:** Sipeng Shen, Ruyang Zhang, Yichen Guo, Elizabeth Loehrer, Yongyue Wei, Ying Zhu, Qianyu Yuan, Sebastian Moran, Thomas Fleischer, Maria M. Bjaanæs, Anna Karlsson, Maria Planck, Johan Staaf, Åslaug Helland, Manel Esteller, Li Su, Feng Chen, David C. Christiani

**Affiliations:** ^1^ Department of Biostatistics School of Public Health Nanjing Medical University China; ^2^ Department of Environmental Health Harvard T.H. Chan School of Public Health Harvard University Boston MA USA; ^3^ China International Cooperation Center of Environment and Human Health Nanjing Medical University China; ^4^ Bellvitge Biomedical Research Institute Institucio Catalana de Recerca i Estudis Avançats University of Barcelona Spain; ^5^ Department of Cancer Genetics Institute of Cancer Research Oslo University Hospital Norway; ^6^ Division of Oncology and Pathology Department of Clinical Sciences Lund Lund University Sweden; ^7^ Institute of Clinical Medicine University of Oslo Norway; ^8^ Key Laboratory of Biomedical Big Data Nanjing Medical University China; ^9^ Pulmonary and Critical Care Unit Massachusetts General Hospital Department of Medicine Harvard Medical School Boston MA USA

**Keywords:** BTG2, early‐stage non‐small cell lung cancer, multi‐omic, prognosis

## Abstract

B‐cell translocation gene 2 (*BTG2*) is a tumour suppressor protein known to be downregulated in several types of cancer. In this study, we investigated a potential role for *BTG2* in early‐stage non‐small cell lung cancer (NSCLC) survival. We analysed *BTG2* methylation data from 1230 early‐stage NSCLC patients from five international cohorts, as well as gene expression data from 3038 lung cancer cases from multiple cohorts. Three CpG probes (cg01798157, cg06373167, cg23371584) that detected *BTG2* hypermethylation in tumour tissues were associated with lower overall survival. The prognostic model based on methylation could distinguish patient survival in the four cohorts [hazard ratio (HR) range, 1.51–2.21] and the independent validation set (HR = 1.85). In the expression analysis, *BTG2* expression was positively correlated with survival in each cohort (HR range, 0.28–0.68), which we confirmed with meta‐analysis (HR = 0.61, 95% CI 0.54–0.68). The three CpG probes were all negatively correlated with *BTG2* expression. Importantly, an integrative model of *BTG2* methylation, expression and clinical information showed better predictive ability in the training set and validation set. In conclusion, the methylation and integrated prognostic signatures based on *BTG2* are stable and reliable biomarkers for early‐stage NSCLC. They may have new applications for appropriate clinical adjuvant trials and personalized treatments in the future.

**Abbreviations**
BTG2: B‐cell translocation gene 2
CI: confidence interval
FDR: false‐discovery rate
GDC: Genomic Data Commons Data Portal
GEO: Gene Expression Omnibus
HR: hazard ratio
LUAD: lung adenocarcinoma
LUSC: lung squamous cell carcinoma
NSCLC: non‐small cell lung cancer
SD: standard deviation

## 1 Introduction

Lung cancer, predominantly non‐small cell lung cancer (NSCLC), which constitutes more than 85% of all lung cancers, is the most commonly diagnosed malignant disease and is a leading cause of cancer‐related deaths worldwide (Chen *et al*., 2014; Wood *et al*., 2016). Diagnosis often occurs in late‐stage disease, when most patients have missed the optimal window for surgery, so prognosis is usually poor. However, genomic profiling of tumour tissues can identify biomarkers for survival prediction of NSCLC and help develop target therapy. Compared with patients diagnosed with late‐stage disease, patients diagnosed with early‐stage disease have a considerably more favourable prognosis, although different prognoses still exist among patients with similar clinical characteristics (Hirsch *et al*., 2017). This phenomenon indicates the importance of improved understanding of genetic and molecular heterogeneity among these patients. In addition to the traditional molecular biomarkers, DNA methylation has improved our understanding of tumour genomics by identifying key biomarkers for multiple cancers and has played an important role in the development of targeted therapy (Bock *et al*., 2016; Jones *et al*., 2016).

Recently, a number of studies have proposed lung cancer signatures for survival stratification with different types of data, including gene expression (Der *et al*., 2014; Shedden *et al*., 2008), DNA methylation (Karlsson *et al*., 2014; Sandoval *et al*., 2013) and microRNA expression (Raponi *et al*., 2009; Tan *et al*., 2011). However, none has been incorporated into clinical practice owing to issues such as lack of sufficient validation, small sample size and overfitting problems. Besides, each proposed signature was limited to only one type of omics data. Robles *et al*. (2015) proposed an integrated prognostic classifier for early‐stage lung cancer, but their results found that different gene biomarkers of methylation and gene expression, when combined with the small sample size, made suggestions for a single target for therapy difficult. A large‐scale multi‐omics data integration is needed for lung cancer to build a cross‐platform prognostic signature.

Two recent studies reported that B‐cell translocation gene 2 (*BTG2*) plays an important role in cancer progression (Dolezal *et al*., 2017; Stupfler *et al*., 2016). *BTG2*, also called *PC3/APRO1/TIS21*, was the first identified gene in BTG/TOB family (Buanne *et al*., 2000). It is located on 1q32.1 and encodes 158 amino acids (Lim, 2006). Several studies have reported that *BTG2* expression is downregulated in some cancers, including laryngeal carcinoma (Liu *et al*., 2009), pancreatic cancer (Coppola *et al*., 2013) and renal cell carcinoma (Struckmann *et al*., 2004). Further*, BTG2* expression has also been found to be related to prognosis in bladder cancer (Wagener *et al*., 2013), breast cancer (Takahashi *et al*., 2011) and pancreatic cancer (Frampton *et al*., 2014). However, the study of *BTG2* in lung cancer has been limited to cell lines (Sun *et al*., 2013; Wei *et al*., 2012). No studies have focused on the role of *BTG2* in lung cancer prognosis, and no Lung Cancer cohort to date has validated its prognostic value.

In this study, using multi‐centre cohorts with methylation and gene expression data, we carried out an integrative study to explore the prognostic role of *BTG2* in early‐stage (clinical stage I, II) NSCLC. The proposed prognostic signatures were successfully validated in all the cohorts and improved the survival prediction ability for early‐stage NSCLC prognosis. In addition, we found *BTG2* had a better prediction performance in cases with adjuvant therapy, which may provide a novel therapeutic target for early‐stage cases.

## 2 Materials and methods

### 2.1 Study populations

#### 2.1.1 Harvard

All patients in the Harvard cohort have been recruited at Massachusetts General Hospital (MGH) from 1992 to present, and all were newly diagnosed, histologically confirmed primary NSCLC at the time of recruitment. Snap‐frozen tumour samples were collected from NSCLC patients during curative surgery with complete resection. Relatively complete survival information was available for the 151 early‐stage patients who were selected for this study. Tumour DNA was extracted from 5‐μm‐thick histopathological sections. Each specimen was evaluated by an MGH pathologist for amount (tumour cellularity > 70%) and quality of tumour cells, and was histologically classified using WHO criteria. The study protocol was approved by the Institutional Review Board of MGH. All patients provided written informed consent.

#### 2.1.2 Sweden

Tumour tissue specimens were collected from early‐stage lung cancer patient who had been operated on at Skane University Hospital, Lund, Sweden (Karlsson *et al*., 2014). The study was approved by the Regional Ethical Review Board in Lund, Sweden (Registration no. 2004/762 and 2008/702). All patients provided written informed consent.

#### 2.1.3 Spain

Descriptions of this study population have been reported previously (Sandoval *et al*., 2013). In brief, tumours were collected by surgical resection from patients who provided consent and with approval from the institutional review boards. The median clinical follow up was 7.2 years. The study was approved by the Bellvitge Biomedical Research Institute institutional review board. All patients provided written informed consent.

#### 2.1.4 Norway

As described previously (Bjaanaes *et al*., 2016), the participants were patients with operable lung cancer tumours who were seen at Oslo University Hospital‐Rikshospitalet, Norway, from 2006 to 2011. Only early‐stage (stage I, II) patients were selected for the current study. The project was approved by the Oslo University institutional review board and regional ethics committee (S‐05307). All patients received oral and written information about the study and signed a written consent before entering the study.

#### 2.1.5 GDC

Genomic Data Commons Data Portal (GDC) resources included 332 early‐stage lung adenocarcinomas (LUAD) and 285 early‐stage squamous cell carcinomas (LUSC) with both survival information and clinical information available for this analysis. In addition, 51 pairs (methylation) and 74 pairs (expression) of early‐stage cases with both tumour and adjacent normal tissue data were used for the differential analysis. Level‐1 HumanMethylation450 DNA methylation data (image data) for each patient were downloaded on 1 October 2015.

**Figure 1:**
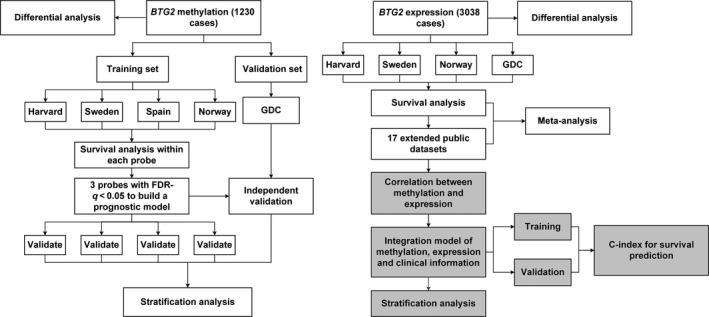
Flow chart indicating study design. The whole study could be divided into three parts. First, we used the methylation data to compare the difference between tumour and normal tissue, build a prognostic model, and validate it in the different cohorts. Secondly, we used the gene expression data to evaluate the BTG2 expression and overall survival by meta‐analysis. Lastly, we performed an integration analysis based on clinical information, methylation and expression data.

The study design is shown in Fig. 1. The data preprocessing details are provided in the Supporting Information. Descriptions of the demographic and clinical characteristics of early‐stage lung cancer patients from the five international study cohorts are shown in Table 1. After data preprocessing, we extracted 13 CpG probes located in the *BTG2* region from the microarray (Table S1), eight in the promoter region and five in the gene body or 3′UTR region.

**Table 1:** Demographic and clinical characteristic descriptions for early‐stage lung cancer patients from five international study cohorts

Characteristics^a^	Cohort 1: Harvard (*n *=* *151)	Cohort 2: Spain (*n *=* *226)	Cohort 3: Norway (*n *=* *133)	Cohort 4: Sweden (*n *=* *103)	Cohort 5: GDC (*n *=* *617)	Overall sample^b^ (*n *=* *1230)
Survival year						
Median (95% CI)	6.66 (5.41–7.87)	7.12 (5.06–9.63)	7.36 (6.77–7.95)^c^	7.39 (4.98–9.12)	4.54 (3.68–5.41)	6.60 (5.84–7.35)
Censored rate, %	19.21	55.31	68.42	43.27	76.99	62.14
Individuals with gene expression data^d^ (%)	26 (17.22)	0 (0)	94 (70.68)	97 (94.17)	613 (99.35)	830 (67.48)
Age (years)	67.67 ± 9.92	65.67 ± 10.58	65.52 ± 9.34	66.45 ± 9.98	66.51 ± 9.47	66.47 ± 9.78
Gender (%)						
Female	67 (44.37)	105 (46.46)	71 (53.38)	54 (52.88)	255 (41.33)	552 (44.92)
Male	84 (55.63)	121 (53.54)	62 (46.62)	49 (47.12)	362 (58.67)	678 (55.08)
Race (%)						
White	151 (100)	226 (100)	133 (100)	103 (100)	488 (79.09)	1101 (89.51)
Black or African‐American	0	0	0	0	57 (9.24)	57 (4.63)
Asian	0	0	0	0	8 (1.30)	8 (0.65)
NA^e^	0	0	0	0	64	64
Smoking status (%)						
Never	18 (11.92)	30 (13.27)	17 (12.78)	18 (17.48)	55 (8.91)	138 (11.42)
Current or former	133 (88.08)	191 (84.51)	116 (87.22)	85 (82.52)	544 (88.17)	1069 (86.91)
NA^e^	0	5	0	0	18	23
Clinical stage (%)						
I	104 (68.87)	183 (80.97)	93 (69.92)	95 (92.31)	393 (63.70)	868 (70.59)
II	47 (31.13)	43 (19.03)	40 (30.08)	8 (7.69)	224 (36.30)	362 (29.41)
Histology (%)						
LUAD	96 (63.58)	183 (80.97)	133 (100.00)	80 (77.88)	332 (53.81)	824 (67.02)
LUSC	55 (36.42)	43 (19.03)	0 (0.00)	23 (22.12)	285 (46.19)	406 (32.98)
Chemotherapy (%)						
No	142 (94.04)	177 (90.77)	102 (76.69)	67 (90.67)	194 (76.98)	682 (84.72)
Yes	9 (5.96)	18 (9.23)	31 (23.31)	7 (9.33)	58 (23.02)	123 (15.28)
NA^e^	0	31	0	29	365	425
Radiotherapy (%)						
No	132 (87.42)	184 (95.13)	132 (99.25)	74 (100.00)	239 (94.84)	761 (96.42)
Yes	19 (12.58)	11 (4.87)	1 (0.75)	0 (0.00)	13 (5.16)	44 (3.58)
NA^e^	0	31	0	29	365	425
Adjuvant therapy (%)						
No	127 (84.11)	168 (86.15)	101 (75.94)	67 (90.54)	187 (74.21)	650 (80.75)
Yes	24 (15.89)	27 (13.85)	32 (24.06)	7 (9.46)	65 (25.79)	155 (19.25)
NA^e^	0	31	0	29	365	425

^a^Cohort 2: Spain is a collaborative study centre including samples from Spain, Italy, UK and France. Adjuvant therapy including chemotherapy or radiotherapy. ^b^DNA methylation 450 Beadchip data are available for all the samples. ^c^The restricted mean survival time was given, as the median was not available. ^d^Specifies the patients for whom both methylation and gene expression data are available. ^e^NA, not available.

#### 2.1.6 Public GEO datasets

We collected 17 extra public datasets of 2209 early‐stage NSCLC gene expression from the Gene Expression Omnibus (GEO) database (Table S2). Cases with data available on survival time, clinical stage and tumour tissue expression values were included.

### 2.2 Statistical analysis

Continuous variables were summarized as mean ± standard deviation (SD), and categorized variables were described by frequency (*n*) and proportion (%). We used a paired Student's *t*‐test to compare the differential methylation/expression values between tumour and adjacent normal tissues. We used a linear model to explore the relationship between different omics data. The false‐discovery rate (FDR) correction *q*‐value was used for multiple comparisons.

We performed meta‐analysis of summary‐level results using an inverse‐variance‐weighted fixed‐effects model with the R package *meta*.

In the survival analysis, associations between *BTG2* CpG probes and overall survival were evaluated by univariable Cox proportional hazard models separately. The methylation prognostic model was calculated as per 1% methylation increments (Shen *et al*., 2017). Kaplan–Meier survival curves were drawn and compared among subgroups using log‐rank tests. In the multivariable Cox regression model, age, gender, clinical stage, smoking status, histology type and study site (if there were two or more sites) were included as covariates. In the integration analysis, the integrated model was built using a multivariable Cox regression model including age, stage, *BTG2* methylation signature and gene expression to generate the coefficients. To evaluate the model prediction accuracy, a concordance statistic (C‐index) was estimated using R package *rms* and compared using R package *compareC* (Kang *et al*., 2015).

Statistical analyses were performed using R version 3.4.0 (The R Foundation). *P*‐values were two‐sided, and *P* (FDR*‐q*)* *< 0.05 was considered statistically significant.

## 3 Results

### 3.1 DNA methylation from BTG2 is associated with lung cancer survival

**Figure 2:**
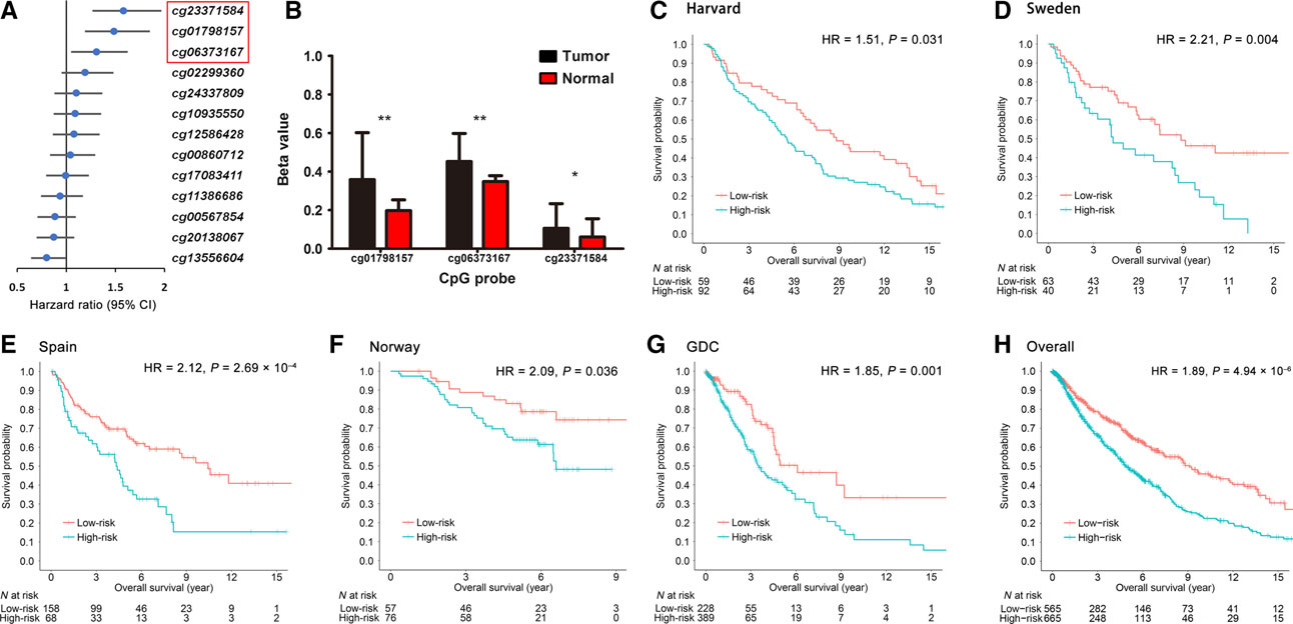
Methylation analysis for BTG2. (A) HR with 95% confidence interval (95% CI) of the 13 CpG sites in Cox regression analysis in the training set. The top three probes were significantly associated with survival. (B) Methylation differential comparison of the three probes between tumour and adjacent normal tissues. Data were described as mean and SD. **FDR‐q < 0.001; *FDR‐q < 0.05. (C–H) Kaplan–Meier survival analyses of the methylation prognostic model, which were categorized into low‐risk and high‐risk groups using a cut‐off value of the median value in the training set for (C) Harvard, (D) Sweden, (E) Spain, (F) Norway, (G) GDC, and (H) overall dataset.

First, we evaluated the association between the 13 CpG probes located in the *BTG2* region and early‐stage NSCLC overall survival in the training set including Harvard, Sweden, Spain and Norway cohorts. Three risk probes were significant with FDR‐*q *< 0.05: cg01798157 (HR = 1.49, 95% CI 1.19–1.85, *q *= 0.002), cg06373167 (HR = 1.31, 95% CI 1.05–1.63, *q *= 0.043) and cg23371584 (HR = 1.58, 95% CI 1.27–1.97, *q *= 6.65 × 10^−4^) (Figs 2A and S1, Table S3). In addition, when we compared the probes between tumour and adjacent normal tissues, the three risk CpG sites were all significantly hypermethylated in tumour tissues (fold change: 1.30–1.82; *q *=* *1.78 × 10^−3^ to 5.03 × 10^−5^) (Fig. 2B, Table S4).

Based on the three survival‐related CpG probes, we built a multi‐loci prognostic model. Using the training set to generate coefficients by Cox regression, the model is: prognostic score_methylation _= 0.0046  × cg01798157 + 0.0026 × cg06373167 + 0.0066 × cg23371584. Increased DNA methylation levels of the three probes were associated with increased risk of death. Patients were divided into high‐risk (above the median) and low‐risk (below the median) groups by the median score in the training set (0.292). We then validated the model separately within each cohort of the training set. Compared with cases in the low‐risk group, cases in the high‐risk group had the worse overall survival in the Harvard (log‐rank test, *P *=* *0.030), Sweden (*P *=* *0.002), Spain (*P *=* *8.71 × 10^−5^) and Norway (*P *=* *0.017) cohorts (Fig. 2C–F). In the multivariable Cox regression model, the score retained significance in the Harvard (HR = 1.51; 95% CI 1.04–2.19; *P* = 0.031), Sweden (HR = 2.21; 95% CI 1.28–3.81; *P *=* *0.004), Spain (HR = 2.12; 95% CI 1.41–3.17; *P *=* *2.69 × 10^−4^) and Norway (HR = 2.09; 95% CI 1.05–4.18; *P* = 0.036) cohorts.

To estimate the reproducibility and validity of the three‐CpG‐based classifier, we performed an independent validation in the GDC cohort. The prognostic score for each patient was calculated with the same formula and divided by the same cut‐off value (0.292) used in the training set. Cases with lower risk scores generally had a better survival than those with higher risk scores (log‐rank test, *P *=* *0.010) (Fig. 2G). After adjusting for the same covariates used in the training set, the methylation model remained an independent prognostic factor (HR = 1.85; 95% CI 1.26–2.72; *P *=* *0.001) (Table S5).

### 3.2 BTG2 gene expression is also associated with survival

**Figure 3:**
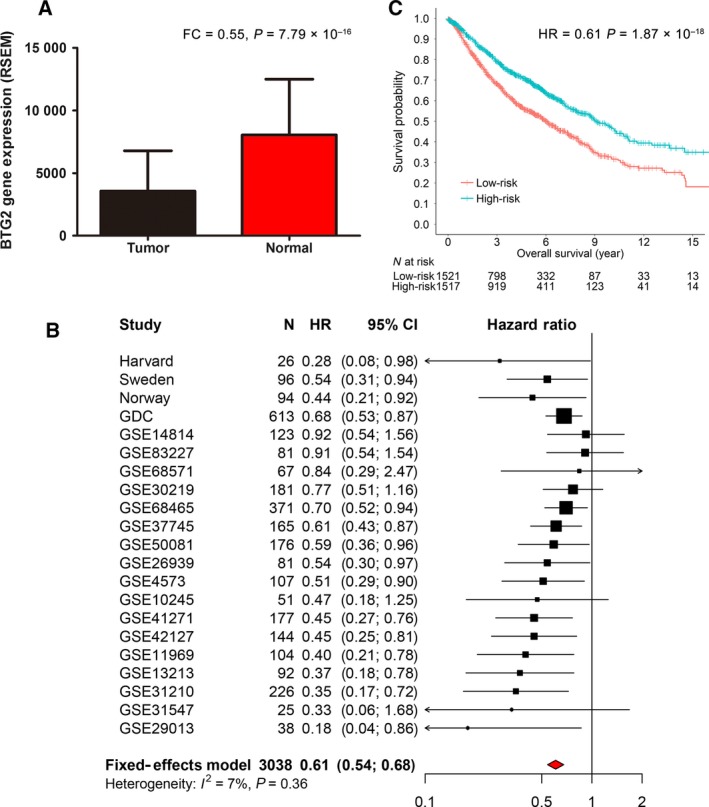
Gene expression analysis for BTG2. (A) BTG2 expression differential analysis between tumour and adjacent normal tissues. Data were described as mean and SD. (B) Meta‐analysis with fixed‐effect model for the BTG2 expression and early‐stage lung cancer survival collected from our cohorts and 17 extended public datasets. (C) Kaplan–Meier survival analyses for the cases in the meta‐analysis. Patients were categorized into low‐risk and high‐risk groups using a cut‐off value of the median value within each cohort.

To compare the *BTG2* expression difference, we extracted 74 early‐stage cases from the GDC cohort with data on both tumour and adjacent normal tissue gene expression. Using a paired Student's *t*‐test, *BTG2* was significantly downregulated in tumour tissues (fold change = 0.55, *P *=* *7.79 × 10^−16^) (Fig. 3A).

Of the five cohorts, gene expression data were available in four cohorts but not in the Spain cohort. In the survival analysis, using the median expression within each cohort as a cut‐off to dichotomize expression levels, *BTG2* over‐expression was significantly associated with better survival in the Harvard (HR = 0.28, *P *=* *0.036), Sweden (HR = 0.54, *P *= 0.023), Norway (HR = 0.44, *P *=* *0.032) and GDC (HR = 0.68, *P *=* *0.005) cohorts (Fig. S2).

Further, we performed a meta‐analysis to examine the relationship between *BTG2* expression and overall survival from the four consortium cohorts and 17 external public lung cancer cohorts. The analysis of these 3038 cases also revealed *BTG2* as a tumour suppressor gene, with higher expression levels associated with longer overall survival (HR = 0.61, 95% CI 0.54–0.68, *P *=* *1.87 × 10^−18^) (Fig. 3B,C). In addition, we also performed a sensitivity analysis using the normalized continuous gene expression data (mean = 0, SD = 1) to test the model robustness. Meta‐analysis also showed that *BTG2* continuous gene expression was significantly associated with overall survival (HR = 0.79; 95% CI 0.74–0.84; *P *=* *2.62 × 10^−13^) (Fig. S3).

### 3.3 Correlation between methylation and expression

**Figure 4:**
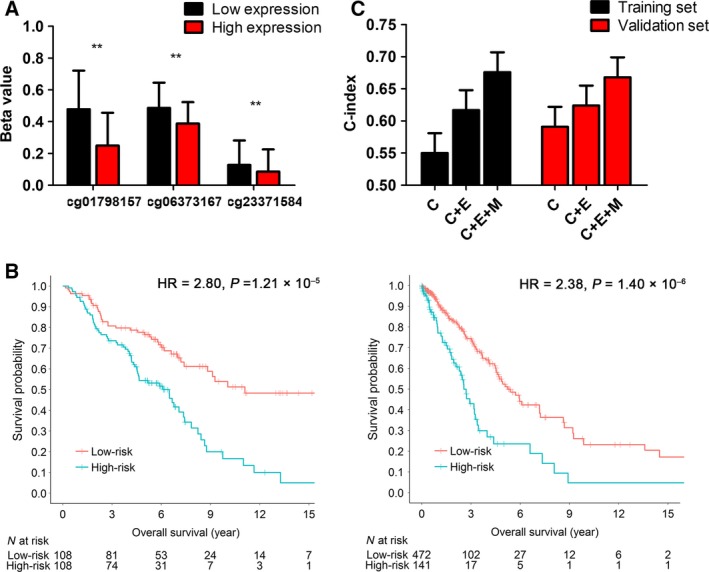
Integration analysis for BTG2. (A) Correlation analysis for the three CpG probes and BTG2 expression using a linear regression model. Expression values within each cohort were dichotomized by the median value and combined. Methylation beta‐values were described as mean and SD. **FDR‐q < 0.001. (B) Kaplan–Meier survival analyses of the integrated prognostic model, which were categorized into low‐risk and high‐risk groups using a cut‐off value of the median value in the training set and validation set. (C) C‐index with standard error bar are shown in the two sets, including clinical information (C), gene expression (E) and methylation (M). The integration model (C + E + M) showed the best predictive performance.

As the mRNA platforms were different, expression values within each cohort were also dichotomized and combined. A linear regression model was used to explore the correlation between methylation and expression in the combined datasets. We found that the three risk CpG probes were all negatively associated with *BTG2* gene expression levels (cg01798157: β = –22.9, 95% CI −26.0 to −19.8, *q *=* *1.52 × 10^−42^; cg06373167: β = −9.8, 95% CI −11.7 to −7.76, *q* = 5.41 × 10^−20^; cg23371584: β = −4.18, 95% CI −6.19 to −2.18, *q *=* *6.90 × 10^−5^) (Fig. 4A).

### 3.4 Integration analysis of clinical information, expression and methylation

To improve the accuracy of clinical prognosis prediction, we performed an integration model for *BTG2* expression, methylation and clinical information. In the multivariate analysis, clinical variables, including age and clinical stage, were independent prognostic factors (Table S5) and were included in the integration model. Expression data were treated as a binary variable (low vs. high). We used a training set using the Harvard, Sweden and Norway cohorts to derive a prognostic score_integration_: 0.027 × age + 0.233 × stage − 0.586 × *BTG2*_mRNA_ + 48.15 × score_methylation model_. Using the the median risk score value of the training sets (2.36) as a cut‐off, the integrated model showed a better ability to distinguish between prognosis compared with the methylation model alone in both the training set (HR = 2.80, 95% CI 1.96–4.28, *P *=* *1.21 × 10^−5^) and the GDC validation cohort (HR = 2.38, 95% CI 1.67–3.37, *P *=* *1.40 × 10^−6^) (Fig. 4B). The integration model also showed a superior predictive performance in comparison with the model using clinical characteristics only (age and clinical stage) (training set C‐index: 0.676 vs. 0.550, *z* = 4.06, *P *=* *4.82 × 10^−5^; validation set C‐index: 0.668 vs. 0.591, *z* = 2.48, *P *=* *0.012) (Fig. 4C).

### 3.5 Stratification analysis for the prognostic signatures

**Figure 5:**
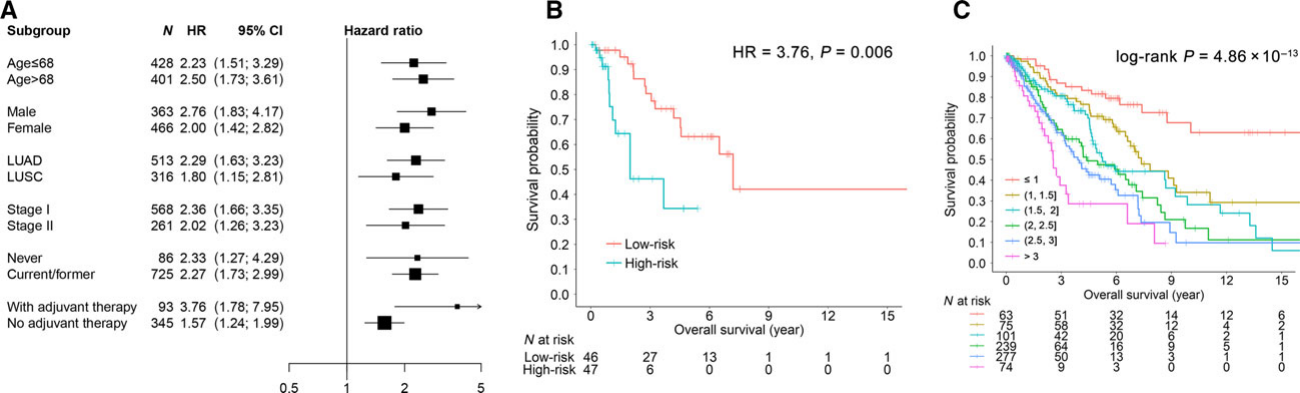
Stratification analysis for the methylation and integration prognostic signatures. (A) HR with 95% CI of overall survival for the overall cases in different subgroups stratified by clinical parameters for the integration model. (B) Kaplan–Meier curves for the cases with adjuvant therapy. (C) Kaplan–Meier curves regarding overall survival for respective different score categories in integration model.

We assessed the effect of methylation and integration prognostic scores on overall survival in subgroups of patients with different clinical profiles. When stratified by clinical variables [age (divided by the median value), gender, histology, clinical stage, smoking status and adjuvant therapy], the models remained statistically significant (Figs 5A and S4A). Interestingly, the effect of the integration signature was more pronounced in patients who received adjuvant therapy (HR = 3.76, 95% CI 1.46–9.68) than in those who did not (HR = 1.57, 95% CI 1.24–1.99) (Fig. 5B).

The Kaplan–Meier curves for overall survival for respective prognostic score categories are shown in Figs 5C and S4B. The classifiers successfully categorized patients into different subgroups with significant differences in clinical outcome (*P*_methylation_ = 1.66 × 10^−7^, *P*_integration_ = 4.86 × 10^−13^).

## 4 Discussion

Early‐stage NSCLC patients are at substantial risk for recurrence and death, even after curative surgical resection. The use of adjuvant therapy in early‐stage disease, particularly for stage I cases, remains controversial because previous randomized trials have not demonstrated a consistent survival benefit (Li *et al*., 2017). Stable and reliable prognostic biomarkers are urgently needed to identify the subgroup at higher risk for death. In this study, we developed prognostic signatures that together with traditional clinical information, DNA methylation and gene expression from only one gene, *BTG2*, are practical for developing targeted therapy. The prognostic signatures could distinguish patient survival and were successfully validated in all cohorts, both in the whole set and in clinically defined subgroups (e.g. stage I, II, and LUAD, LUSC). The integrated model could add prognostic predictive value to the clinical information currently available.

*BTG2* is one of the early growth response genes (Sukhatme *et al*., 1987) and is highly expressed in multiple organs and tissues, including lung, intestines, pancreas and prostate (Melamed *et al*., 2002). Several cancer‐related biological functions have been found in this gene. First, over‐expression of *BTG2* is known to inhibit proliferation of cells and invasion in some tumours, including lung cancer cells (Wei *et al*., 2012), and acts as an anti‐proliferation gene in cooperation with *PRMT1* (Dolezal *et al*., 2017). Secondly, *BTG2* is involved in the development and differentiation of cancer cells that could promote retinoic acid‐induced differentiation in haematopoietic cells (Passeri *et al*., 2006). Thirdly, a previous study has reported that *BTG2* was able to promote or induce cell apoptosis and suppress cell invasion in triple‐negative breast cancer cells (Zhang *et al*., 2013). Fourthly, *BTG2* is one of the p53 target genes and is involved in the DNA damage repair process. It acts through the p53‐dependent Ras signal transduction pathway and significantly increases expression when DNA is damaged (Boiko *et al*., 2006). Thus, *BTG2* plays important roles in cell proliferation, differentiation, apoptosis and DNA damage repair.

**Figure 6:**
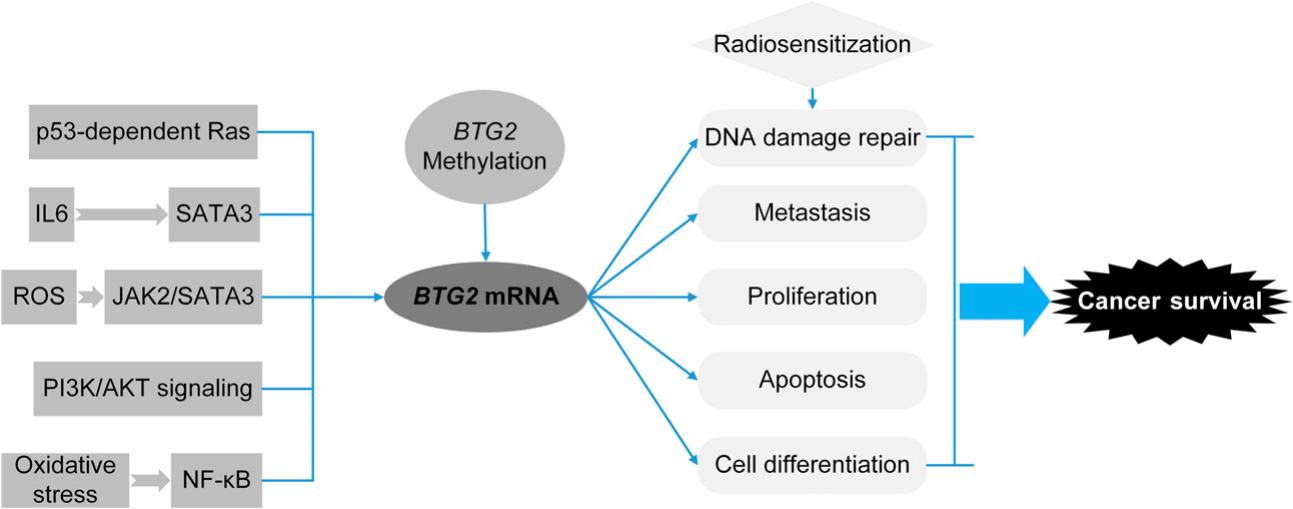
Flowchart for BTG2‐involved pathways and biological mechanisms.

*BTG2* is involved in several important cancer‐related pathways (Fig. 6). As described previously, it is a major downstream anti‐activity effector in the p53‐dependent Ras pathway and is linked to the p53 pathway in human tumorigenesis (Boiko *et al*., 2006). Additionally, it inhibits the proliferation and metastasis of cancer cells by suppressing the PI3K/AKT pathway, which is an important pathway involved in the malignant progression of various tumours and mediates the cancer proliferation, migration and invasion (Li *et al*., 2015). Moreover, *BTG2* over‐expression inhibits interleukin‐6 (IL‐6) expression through downregulation in the STAT3 pathway, as well as inhibiting reactive oxygen species (ROS) generation in the JAK2‐STAT3 signalling pathway (Quy *et al*., 2013); thus it has a negative effect on cancer cell growth. *BTG2* expression is also upregulated by oxidative stress via the ROS‐protein kinase C‐ΝFκΒ pathway, which is independent of p53 status (Imran and Lim, 2013). Hence, *BTG2* participates in some pathways that are crucial for cancer development and progression.

As *BTG2* has been reported to relate to cancer via various biological mechanisms, it has a potential to be a target gene for precision treatment. In our stratification analysis, we found that the prognostic signature was more effective and had a better 5‐year prediction performance in patients who received adjuvant therapy than in those who did not. In terms of clinical application, *BTG2* has been demonstrated to be one of the hypoxia‐inducible proapoptotic targets of p53, which can modulate apoptosis and radiosensitivity via AKT inhibition (Leszczynska *et al*., 2015). Further, previous reports suggest that *BTG2* expression improved the radiosensitivity of NSCLC and breast cancer cells by affecting cell cycle distribution, enhancing radiation‐induced apoptosis and inhibiting DNA repair‐related protein expression (He *et al*., 2015; Hu *et al*., 2012), which suggests that *BTG2* may be a novel target in radiotherapy for lung cancer. Whether *BTG2* plays a role in chemosensitivity still needs further investigation.

We notice that the three risk CpG probes in the methylation prognostic model were all in the gene body or 3′UTR region, whereas most probes in the promoter region were not associated with survival. Recent studies have found that gene body methylation can also alter gene expression, with the genes serving as therapeutic targets (Ball *et al*., 2009; Jones, 2012; Yang *et al*., 2014), e.g. *ITPKA* (Wang *et al*., 2016). In addition, the three probes showed a strong negative correlation with *BTG2* expression. Thus, the proposed epigenetic silencing CpG probes might be important regulators of gene expression.

To our knowledge, this is the first multi‐centre, large‐scale integration analysis of *BTG2* methylation and expression in early‐stage NSCLC. We acknowledge some limitations. First, the sample size for some subgroups, such as patients with radiotherapy, was not large, which made some subgroup analyses difficult to perform. Instead, we chose to analyse cases with some form of adjuvant therapy. Secondly, the histological subtypes in the five cohorts were not in equilibrium. Specifically, no LUSC cases were included in the Norway cohort. However, the prognostic signatures we identified were significant in both major histological subtypes, reducing concerns of bias. Thirdly, the scope of this study is limited when compared with other whole‐genome level studies.

## 5 Conclusions

The proposed methylation and integration signatures based on *BTG2* are stable and reliable prognostic biomarkers for early‐stage NSCLC overall survival. These prognostic signatures may have new applications for appropriate adjuvant trials and personalized treatments in the future.

## Author contributions

SS, RZ, DCC and FC contributed to the study design. RZ, SM, AH, MMB, AK, MP, ME, TF, JS and LS contributed to data collection. SS, YG and YW performed statistical analysis and interpretation. SS drafted the manuscript. SS, EL, YZ and QY revised the manuscript. DCC obtained funding for the study. All authors contributed to critical revision of the final manuscript and approved the final version of the manuscript. Sponsors had no role in study design, data collection and analysis, decision to publish, or preparation of the manuscript.

## Consent for publication

All participants gave written informed consent. All authors have reviewed the manuscript and given their consent for publication.

## Web resources

GDC: https://portal.gdc.cancer.gov/

GEO: https://www.ncbi.nlm.nih.gov/gds/

R: https://www.r-project.org/

## Supporting information


**Fig. S1.** Boxplot depicting the distribution of the three CpG probes across the five cohorts.
**Fig. S2.** (A) Sweden, (B) Norway, (C) Harvard and (D) GDC.
**Fig. S3.** Meta‐analysis with fixed‐effect model for the *BTG2* expression and early‐stage lung cancer survival collected from our cohorts and 17 extended public datasets.
**Fig. S4.** (A) Stratification analysis for prognostic signature based on methylation model. (B). Kaplan–Meier curves regarding overall survival for respective different score categories in the methylation model.
**Table S1.** Annotation for 13 CpG sites located in *BTG2* gene region.
**Table S2.** Study characteristics of the 17 public lung cancer datasets.
**Table S3.** Cox regression analysis for the 13 probes in the training set.
**Table S4.** Differential analysis between tumour and adjacent normal tissues for the 13 probes.
**Table S5.** Multivariable Cox regression analysis for the methylation prognostic signature.
