# Supplementary material for: A multi‐omic study reveals BTG2 as a reliable prognostic marker for early‐stage non‐small cell lung cancer
Source: Mol Oncol. 2018 May 4;12(6):913–24. doi: 10.1002/1878-0261.12204 (PMC5983115; doi:10.1002/1878-0261.12204)
Supplement: Supplementary file 1 — Fig. S1. Boxplot depicting the distribution of the three CpG probes across the five cohorts. Fig. S2. (A) Sweden, (B) Norway, (C) Harvard and (D) GDC. Fig. S3. Meta‐analysis with fixed‐effect model for the BTG2 expression and early‐stage lung cancer survival collected from our cohorts and 17 extended public datasets. Fig. S4. (A) Stratification analysis for prognostic signature based on methylation model. (B). Kaplan–Meier curves regarding overall survival for respective different score categories in the methylation model. Table S1. Annotation for 13 CpG sites located in BTG2 gene region. Table S2. Study characteristics of the 17 public lung cancer datasets. Table S3. Cox regression analysis for the 13 probes in the training set. Table S4. Differential analysis between tumour and adjacent normal tissues for the 13 probes. Table S5. Multivariable Cox regression analysis for the methylation prognostic signature. [file MOL2-12-913-s001.pdf]

A multi-omic study reveals *BTG2* as a reliable prognostic marker for early-stage non-small cell lung cancer

Supporting information

DNA methylation profiles

Genome-wide DNA methylation data from each cohort was profiled using Illumina Infinium HumanMethylation450 BeadChip Assay. Raw data were processed using R package *minfi* version 1.22.0 (1). Background subtraction, quantile normalization, and quality control were performed subsequently. Low-quality probes were removed if they met the following criteria: (i) failed detection ($P > 0.05$) in ≥5% samples; (ii) coefficient of variance (CV) <5%; (iii) methylated or unmethylated in all samples; (iv) single nucleotide polymorphisms (SNPs) located in the assayed CpG dinucleotide (2). Samples with >5% undetectable probes also were excluded. BMIQ normalization was used for further type I and II probe correction (3). Further, ComBat (4) was used to adjust batch effects among different cohorts using R package *sva*.

Gene expression profiles

Harvard The mRNA expression data were available from a subset of NSCLC patients. We used the Whole-Genome DASL HT Assay to get the gene expression values. Expression of all genes was normalized using dChip software before analysis.

Norway A subset of LUAD samples had both methylation and mRNA expression data available. The mRNA expression analysis was assessed using

gene expression microarrays from Agilent technologies (SurePrint G3 human GE, 8 x 60 K). The gene expression data was log 2 transformed and normalized between arrays by using the 75th percentile method in Genespring GX analysis Software v.12.1 (Agilent technology).

Sweden Gene expression analysis was performed on 117 tumors using Illumina Human HT-12 V4 microarrays. 97 cases had both methylation and expression data. Gene expression data were quantile normalized and mean-centered for each probe across all samples. Probe sets without signal intensity above the median of negative control intensity signals in at least 80% of samples were excluded from analysis.

GDC GDC RNA sequencing (RNA-Seq) data preprocessing were done by the The Cancer Genome Atlas (TCGA) workgroup. Raw counts were normalized using RNA-Sequencing by Expectation Maximization (RSEM). Level-3 (gene level) gene quantification data were downloaded from GDC data portal and were further checked for quality. Expression of all genes was extracted and quantile normalized before analysis.

17 public datasets We collected 17 public datasets of early-stage NSCLC from the Gene Expression Omnibus (GEO) database. Due the mRNA platforms were different, the expression values were dichotomized into high-expression and low-expression with a cutoff of median value from each cohort.

Reference

1. Aryee MJ. Minfi: a flexible and comprehensive Bioconductor package for the analysis of Infinium DNA methylation microarrays. Bioinformatics. 2014;30(10):1363-9.
2. Sandoval J, Mendezgonzalez J, Nadal E, Chen G, Carmona FJ, Sayols S, et al. A prognostic DNA methylation signature for stage I non-small-cell lung cancer. Journal of Clinical Oncology Official Journal of the American Society of Clinical Oncology. 2013;31(32):4140-7.
3. Teschendorff AE, Marabita F, Lechner M, Bartlett T, Tegner J, Gomez-Cabrero D, et al. A beta-mixture quantile normalization method for correcting probe design bias in Illumina Infinium 450 k DNA methylation data. Bioinformatics. 2013;29(2):189-96.
4. Johnson WE, Li C, Rabinovic A. Adjusting batch effects in microarray expression data using empirical Bayes methods. Biostatistics. 2007;8(1):118-27.

Supplementary Tables and Figures

Table S1. Annotation for 13 CpG sites located in *BTG2* gene region

CpG site	CHR	Position	UCSC_RefGene_Group	Relation_to_UCSC_CpG_Island	Included in the prognostic model
cg20138067	1	203273571	TSS1500	N_Shore	No
cg00567854	1	203273693	TSS1500	N_Shore	No
cg00860712	1	203274134	TSS1500	N_Shore	No
cg12586428	1	203274421	TSS1500	Island	No
cg11386686	1	203274497	TSS200	Island	No
cg17083411	1	203274503	TSS200	Island	No
cg10935550	1	203274660	TSS200	Island	No
cg13556604	1	203274688	5'UTR;1stExon	Island	No
cg24337809	1	203274882	Body	Island	No
cg02299360	1	203275326	Body	Island	No
cg23371584	1	203275927	Body	S_Shore	Yes
cg01798157	1	203276595	3'UTR	S_Shore	Yes
cg06373167	1	203278044	3'UTR	S_Shelf	Yes

Genome build version: GRCh37/hg19.

Table S2. Study characteristics of the 17 public lung cancer datasets

Study ID	Study description	Platform	Probe name	URL
GSE14814	Prognostic and predictive gene signature for adjuvant chemotherapy in resected non-small-cell lung cancer	Affymetrix Human Genome U133A Array	201236_s_at	https://www.ncbi.nlm.nih.gov/geo/query/acc.cgi?acc=GSE14814
GSE4573	Gene expression signatures for predicting prognosis of squamous cell and adenocarcinomas of the lung	Affymetrix Human Genome U133A Array	201236_s_at	https://www.ncbi.nlm.nih.gov/geo/query/acc.cgi?acc=GSE4573
GSE68465	Gene expression-based survival prediction in lung adenocarcinoma: a multi-site, blinded validation study	Affymetrix Human Genome U133A Array	201236_s_at	https://www.ncbi.nlm.nih.gov/geo/query/acc.cgi?acc=GSE68465
GSE31547	MSKCC-A Primary Lung Cancer Specimens	Affymetrix Human Genome U133A Array	201236_s_at	https://www.ncbi.nlm.nih.gov/geo/query/acc.cgi?acc=GSE31547
GSE37745	Biomarker discovery in non-small cell lung cancer: integrating gene expression profiling, meta-analysis, and tissue microarray validation	Affymetrix Human Genome U133 Plus 2.0 Array	201236_s_at	https://www.ncbi.nlm.nih.gov/geo/query/acc.cgi?acc=GSE37745
GSE30219	Ectopic activation of germline and placental genes identifies aggressive metastasis-prone lung cancers	Affymetrix Human Genome U133 Plus 2.0 Array	201236_s_at	https://www.ncbi.nlm.nih.gov/geo/query/acc.cgi?acc=GSE30219
GSE10245	Gene expression differences between adenocarcinoma and squamous cell carcinoma in human NSCLC	Affymetrix Human Genome U133 Plus 2.0 Array	201236_s_at	https://www.ncbi.nlm.nih.gov/geo/query/acc.cgi?acc=GSE10245
GSE50081	Validation of a histology-independent prognostic gene signature for early-stage, non-small-cell lung cancer including stage IA patients	Affymetrix Human Genome U133 Plus 2.0 Array	201236_s_at	https://www.ncbi.nlm.nih.gov/geo/query/acc.cgi?acc=GSE50081
GSE31210	Identification of genes upregulated in ALK-positive and EGFR/KRAS/ALK-negative lung adenocarcinomas	Affymetrix Human Genome U133 Plus 2.0 Array	201236_s_at	https://www.ncbi.nlm.nih.gov/geo/query/acc.cgi?acc=GSE31210
GSE29013	Robust gene expression signature from formalin-fixed paraffin-embedded samples predicts prognosis of non-small-cell lung cancer patients	Affymetrix Human Genome U133 Plus 2.0 Array	201236_s_at	https://www.ncbi.nlm.nih.gov/geo/query/acc.cgi?acc=GSE29013
GSE11969	Expression Profile-Defined Classification of Lung Adenocarcinoma	Agilent Homo sapiens 21.6K custom array	A_23_P62901	https://www.ncbi.nlm.nih.gov/geo/query/acc.cgi?acc=GSE11969
GSE13213	Relapse-related molecular signature in lung adenocarcinomas identifies patients with dismal prognosis	Agilent-014850 Whole Human Genome Microarray 4x44K G4112F	A_23_P62901	https://www.ncbi.nlm.nih.gov/geo/query/acc.cgi?acc=GSE13213

GSE26939	Human lung adenocarcinoma mRNA expression and gene mutations	Agilent-UNC-custom-4X44K	NM_006763_2_2532	https://www.ncbi.nlm.nih.gov/geo/query/acc.cgi?acc=GSE26939
GSE41271	Expression profiling of 275 lung cancer specimens	Illumina HumanWG-6 v3.0 expression beadchip	ILMN_1770085	https://www.ncbi.nlm.nih.gov/geo/query/acc.cgi?acc=GSE41271
GSE42127	Expression data for non-small-cell lung cancer	Illumina HumanWG-6 v3.0 expression beadchip	ILMN_1770085	https://www.ncbi.nlm.nih.gov/geo/query/acc.cgi?acc=GSE42127
GSE83227	Classification of human lung carcinomas by mRNA expression profiling reveals distinct adenocarcinoma subclasses	Affymetrix Human Genome U95 Version 2 Array	36634_at	https://www.ncbi.nlm.nih.gov/geo/query/acc.cgi?acc=GSE83227
GSE68571	Gene-expression profiles predict survival of patients with lung adenocarcinoma	Affymetrix Human Full Length HuGeneFL Array	Y09943_s_at	https://www.ncbi.nlm.nih.gov/geo/query/acc.cgi?acc=GSE68571

Table S3. Cox regression analysis for the 13 probes in the training set

CpG site	Harvard		Sweden		Spain		Norway		Overall		
	HR (95% CI)	*P*	HR (95% CI)	*P*	HR (95% CI)	*P*	HR (95% CI)	*P*	HR (95% CI)	*P*	FDR-*q*
cg20138067	0.72 (0.51-1.03)	0.076	0.71 (0.42-1.19)	0.192	1.35 (0.91-2)	0.136	0.53 (0.28-1)	0.050	0.87 (0.7-1.08)	0.209	0.453
cg00567854	0.78 (0.54-1.11)	0.162	0.71 (0.42-1.2)	0.201	1.26 (0.85-1.87)	0.240	0.57 (0.3-1.06)	0.077	0.88 (0.71-1.1)	0.254	0.472
cg00860712	0.99 (0.69-1.42)	0.966	0.95 (0.56-1.59)	0.836	1.03 (0.7-1.53)	0.873	1.69 (0.91-3.14)	0.094	1.04 (0.83-1.29)	0.732	0.793
cg12586428	1.18 (0.83-1.68)	0.364	0.77 (0.45-1.29)	0.314	1.54 (1.04-2.29)	0.032	1.67 (0.9-3.11)	0.104	1.08 (0.86-1.34)	0.515	0.644
cg11386686	1.48 (1.03-2.12)	0.033	1.2 (0.72-2.01)	0.491	1.15 (0.78-1.7)	0.478	1.11 (0.61-2.04)	0.734	0.93 (0.74-1.17)	0.545	0.644
cg17083411	0.96 (0.67-1.37)	0.811	0.66 (0.39-1.12)	0.123	1.32 (0.89-1.95)	0.163	1.03 (0.56-1.88)	0.934	0.99 (0.79-1.23)	0.920	0.920
cg10935550	1 (0.7-1.42)	0.987	1.12 (0.67-1.89)	0.665	1.57 (1.06-2.34)	0.024	1.17 (0.64-2.15)	0.602	1.09 (0.87-1.36)	0.455	0.644
cg13556604	0.81 (0.57-1.16)	0.256	1.03 (0.61-1.72)	0.915	1.18 (0.8-1.74)	0.407	0.78 (0.42-1.44)	0.424	0.8 (0.64-0.99)	0.043	0.141
cg24337809	1.44 (1.01-2.06)	0.045	0.82 (0.49-1.38)	0.455	1.16 (0.79-1.72)	0.455	1.13 (0.62-2.09)	0.683	1.1 (0.88-1.37)	0.406	0.644
cg02299360	0.99 (0.69-1.41)	0.945	0.85 (0.5-1.42)	0.529	1.78 (1.2-2.65)	0.004	1.24 (0.68-2.28)	0.483	1.19 (0.95-1.48)	0.128	0.333
cg23371584	1.41 (0.98-2.01)	0.061	1.79 (1.05-3.05)	0.031	1.75 (1.18-2.6)	0.006	1.35 (0.73-2.49)	0.333	1.58 (1.27-1.97)	<0.001	<0.001
cg01798157	1.22 (0.85-1.74)	0.275	2.42 (1.4-4.18)	0.001	1.31 (0.89-1.94)	0.175	1.64 (0.88-3.05)	0.117	1.49 (1.19-1.85)	<0.001	0.002
cg06373167	0.81 (0.57-1.16)	0.248	2.16 (1.26-3.7)	0.005	1.39 (0.94-2.06)	0.097	1.38 (0.75-2.55)	0.299	1.31 (1.05-1.63)	0.001	0.043

Each probe was categorized into high- and low-methylated by the median value within each cohort.

Table S4. Differential analysis between tumor and adjacent normal tissues for the 13 probes

CpG site	Beta value (tumor)	Beta value (normal)	Fold change	*P*	FDR-*q*	Trend
cg20138067	0.600	0.637	0.942	1.19E-02	2.21E-02	Down
cg00567854	0.719	0.689	1.043	8.82E-02	1.27E-01	None
cg00860712	0.233	0.318	0.732	1.82E-07	2.37E-06	Down
cg12586428	0.004	0.003	1.317	1.85E-01	2.18E-01	None
cg11386686	0.006	0.005	1.129	6.26E-01	6.78E-01	None
cg17083411	0.233	0.264	0.883	7.77E-04	2.02E-03	Down
cg10935550	0.014	0.010	1.450	1.05E-03	2.27E-03	Up
cg13556604	0.005	0.004	1.076	6.98E-01	6.98E-01	None
cg24337809	0.023	0.011	2.085	1.80E-01	2.18E-01	None
cg02299360	0.058	0.029	2.034	1.76E-02	2.86E-02	Up
cg23371584	0.105	0.060	1.758	5.47E-04	1.78E-03	Up
cg01798157	0.358	0.197	1.819	2.59E-05	1.12E-04	Up
cg06373167	0.452	0.348	1.299	7.74E-06	5.03E-05	Up

Table S5. Multivariable Cox regression analysis for the methylation prognostic signature

Characteristics	Harvard		Sweden		Spain		Norway		GDC		Overall[a]	
	HR (95% CI)	*P*	HR (95% CI)	*P*	HR (95% CI)	*P*	HR (95% CI)	*P*	HR (95% CI)	*P*	HR (95% CI)	*P*
Prognostic score (high risk group)	1.51 (1.04-2.19)	0.031	2.21 (1.28-3.81)	0.004	2.12 (1.41-3.17)	2.69×10^{-4}	2.09 (1.05-4.18)	0.036	1.85 (1.26-2.72)	0.001	1.89 (1.32-2.57)	4.94×10^{-6}
Age (per year)	1.05 (1.03-1.07)	1.57×10^{-5}	1.05 (1.02-1.08)	0.004	0.99 (0.97-1.01)	0.389	1.01 (0.98-1.05)	0.546	1.03 (1.01-1.05)	0.001	1.02 (1.01-1.03)	7.62×10^{-5}
Gender (female)	1.02 (0.69-1.5)	0.915	0.78 (0.43-1.38)	0.388	1.41 (0.91-2.17)	0.126	0.78 (0.42-1.45)	0.429	0.79 (0.54-1.15)	0.220	1.00 (0.83-1.22)	0.963
Clinical stage (stage II)	2.05 (1.35-3.1)	7.10×10^{-4}	1.07 (0.38-3.06)	0.892	2.93 (1.86-4.6)	3.40×10^{-6}	2.17 (1.17-4.03)	0.014	1.41 (0.98-2.03)	0.064	1.90 (1.56-2.34)	2.09×10^{-9}
Smoking status (current smoker)	1.41 (1.04-1.93)	0.029	2.24 (0.91-5.54)	0.080	0.91 (0.47-1.76)	0.783	0.63 (0.24-1.62)	0.336	1 (0.46-2.16)	0.993	1.31 (0.93-1.83)	0.114
Histology type (LUSC)	1.15 (0.78-1.7)	0.487	0.87 (0.42-1.77)	0.692	1.37 (0.84-2.23)	0.201	-	-	1.13 (0.78-1.65)	0.516	1.20 (0.96-1.49)	0.110

[a]To control the potential heterogeneity caused by different geographic regions, study sites were included as a covariate in the multivariable model.

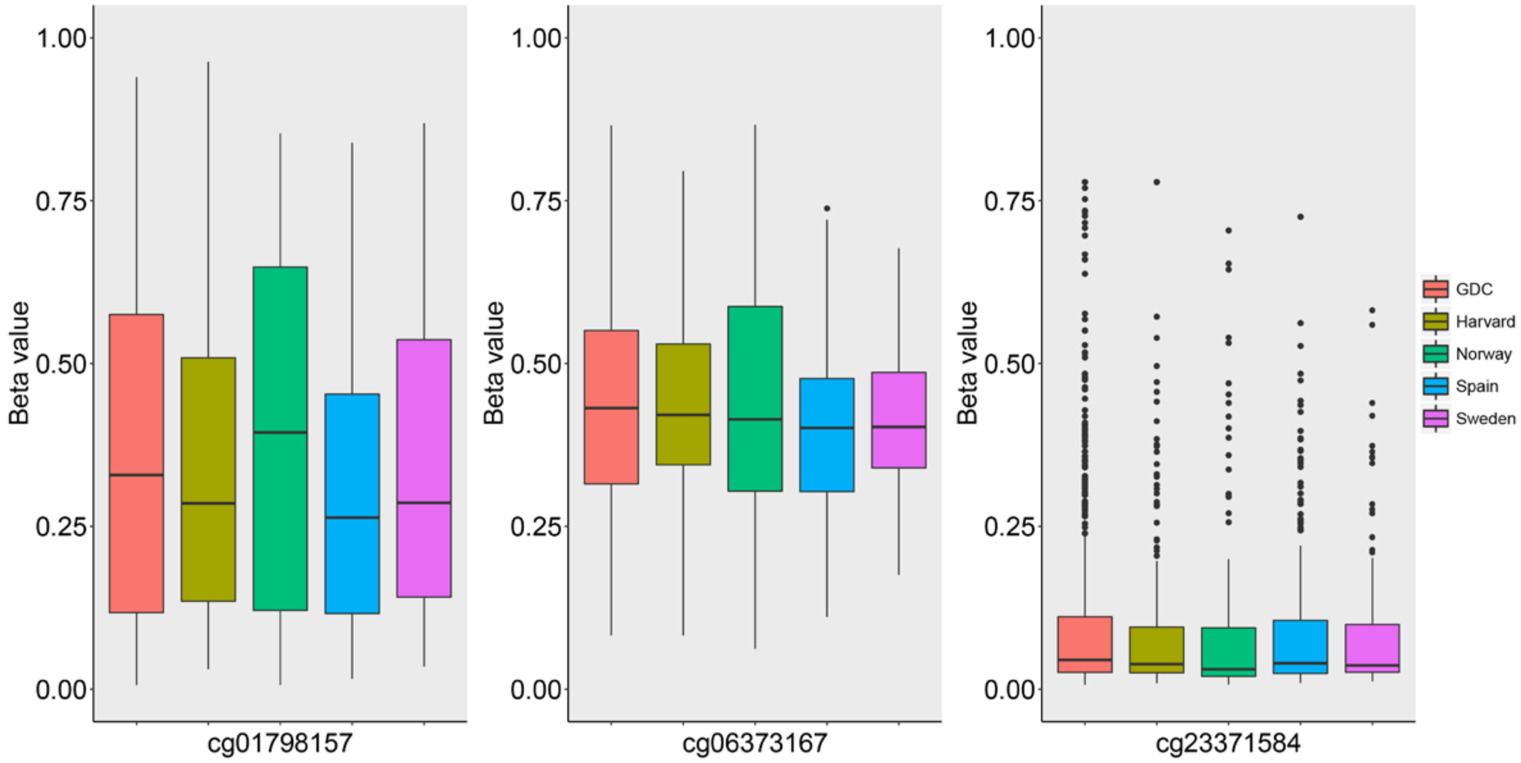

Figure S1. Boxplot depicting the distribution of the three CpG probes across the five cohorts. The central rectangle spans the first quartile to the third quartile (the interquartile range or IQR). A segment inside the rectangle shows the median and "whiskers" above and below the box show the locations of the minimum and maximum.

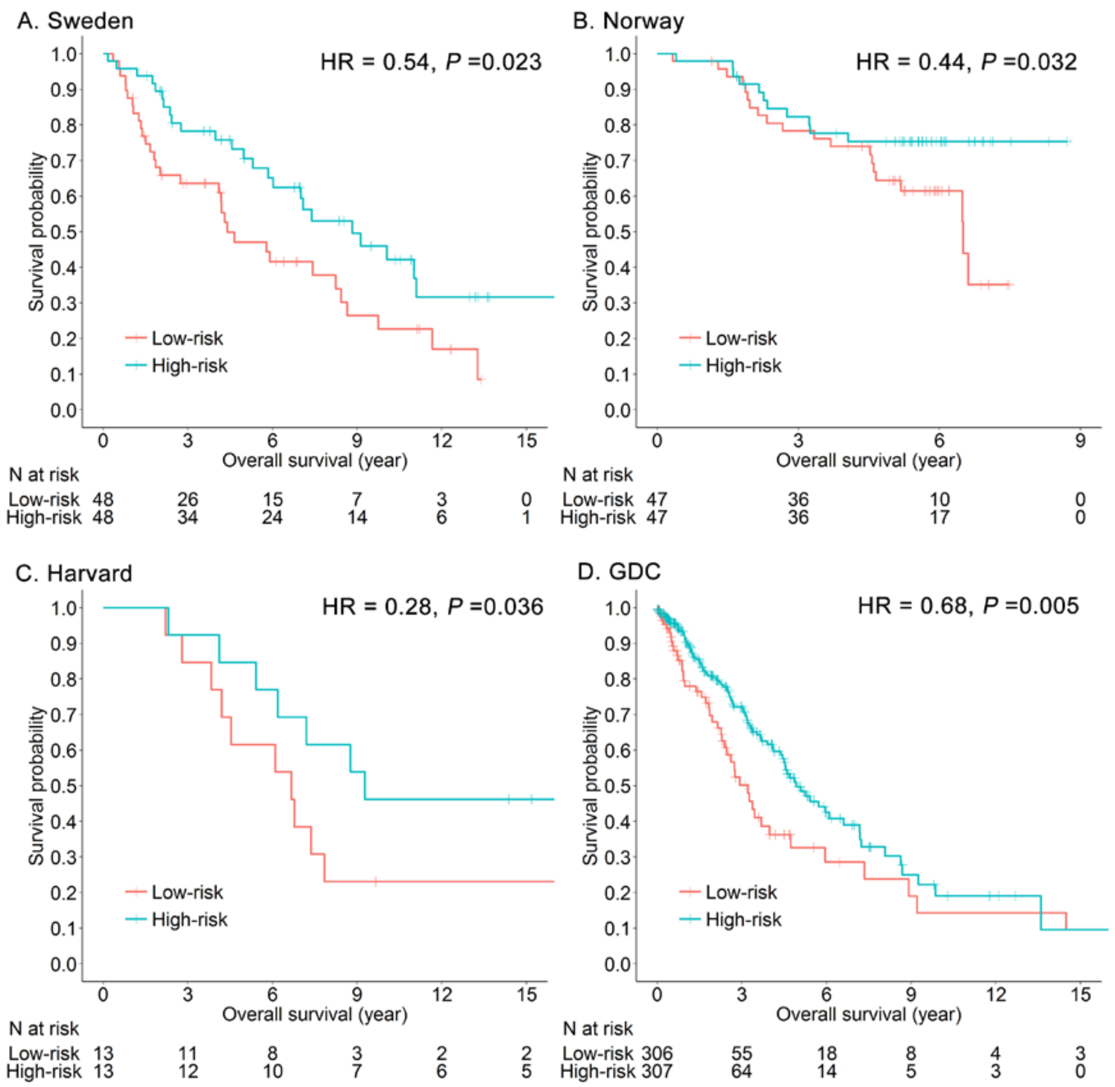

Figure S2. (**A**) Sweden, (**B**) Norway, (**C**) Harvard, and (**D**) GDC. Kaplan-Meier survival analyses for the *BTG2* gene expression in the four cohorts. Patients were categorized into low-risk and high-risk groups using a cutoff value of the median value within each cohort.

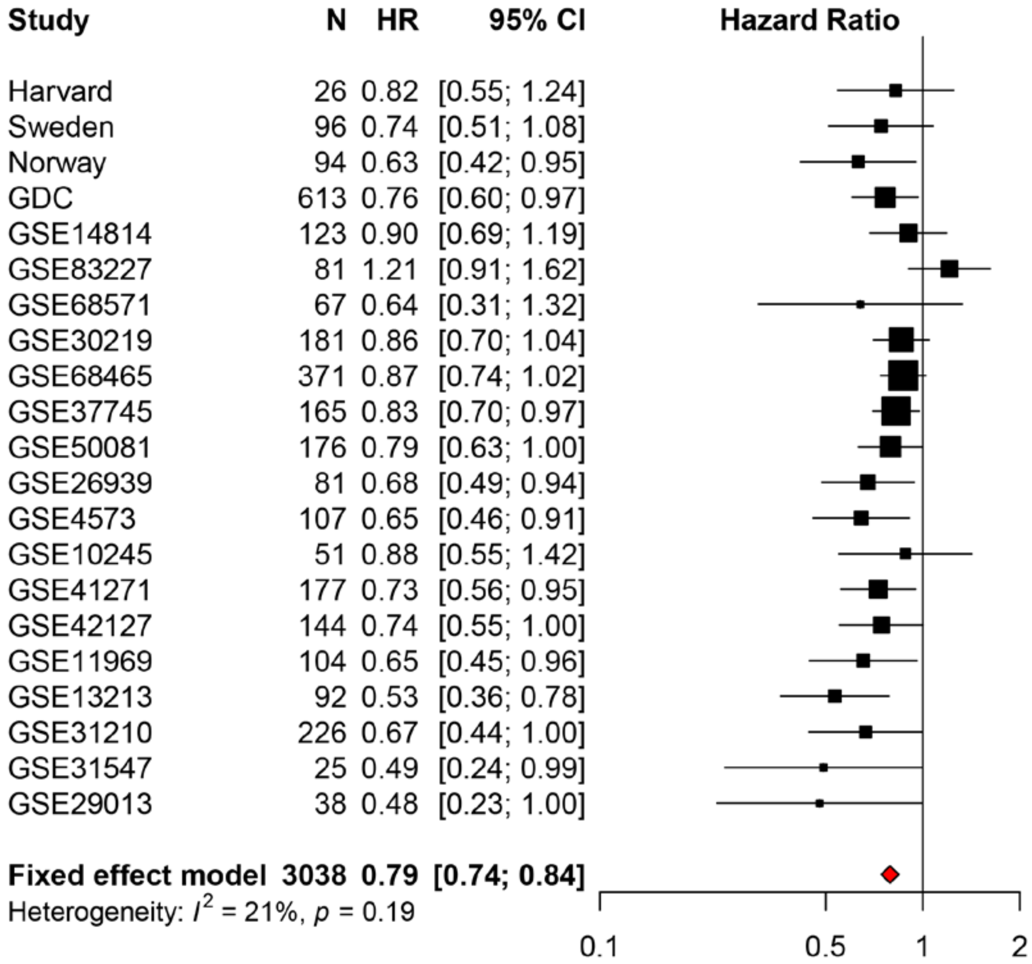

Figure S3. Meta-analysis with fix-effect model for the BTG2 expression and early-stage lung cancer survival collected from our cohorts and 17 extended public datasets. The gene expression data of each cohort was normalized with mean = 0 and standard deviation = 1 and included in the univariable Cox regression model.

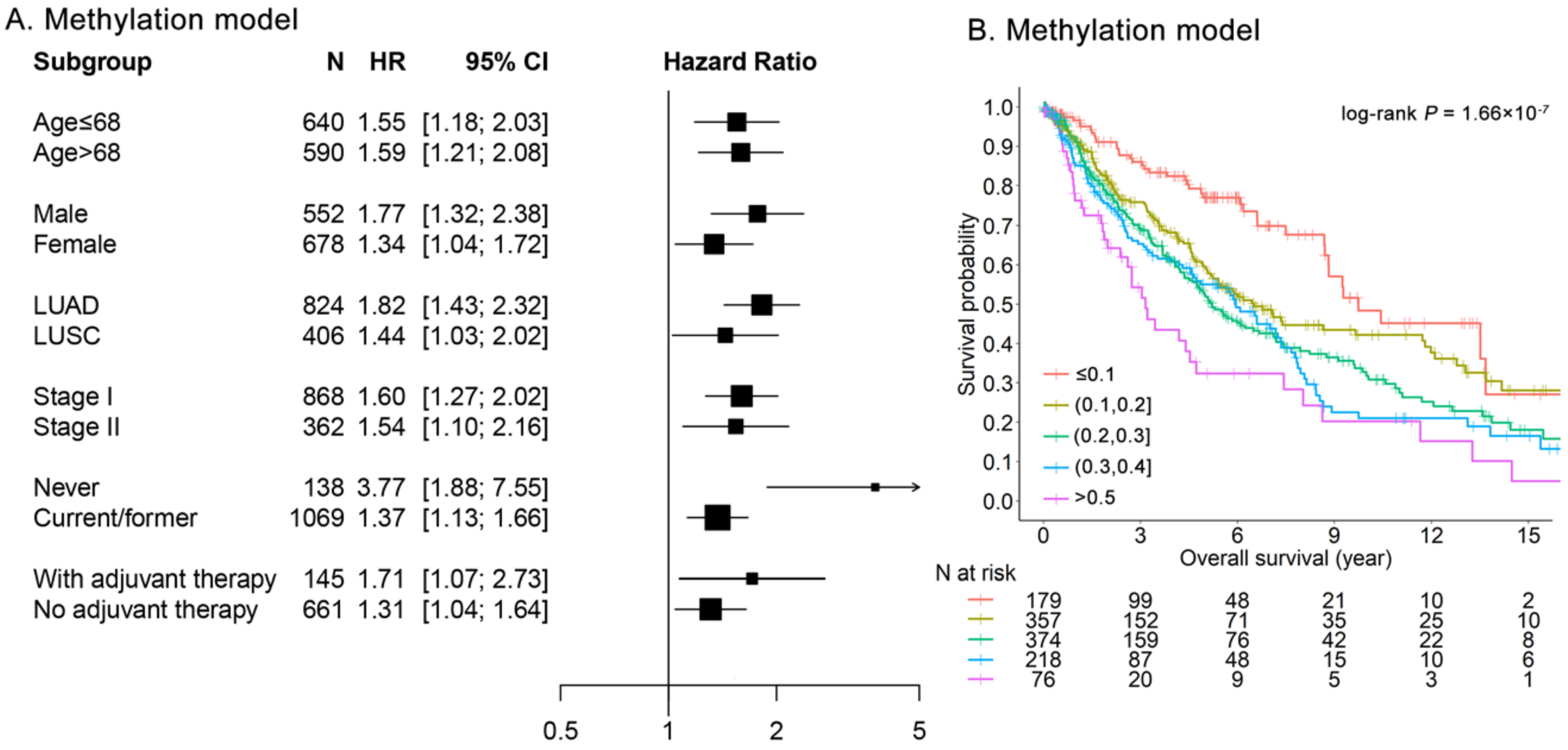

Figure S4. (A) Stratification analysis for prognostic signature based on methylation model. (B). Kaplan–Meier curves regarding overall survival for respective different score categories in the methylation model.
